# Frequency of de novo atrial fibrillation in patients presenting with acute ischemic cerebrovascular stroke

**DOI:** 10.1186/s43044-020-00050-8

**Published:** 2020-04-07

**Authors:** Mahmoud Abdelnabi, Abdallah Almaghraby, Yehia Saleh, Özge Özden Tok, Tuğba Kemaloğlu Öz, Ola Abdelkarim, Haitham Badran

**Affiliations:** 1 Cardiology and Angiology Unit, Department of Clinical and Experimental Internal Medicine, Medical Research Institute, Alexandria University, Alexandria, Egypt; 2 Department of Cardiology, Faculty of Medicine, Alexandria University, Alexandria, Egypt; 3 Michigan State University, East Lansing, Michigan United States of America; 4 Memorial Bahçelievler Hospital, Istanbul, Turkey; 5 Liv Hospital Ulus, Istanbul, Turkey; 6 Department of Cardiology, Faculty of Medicine, Ain Shams University, Cairo, Egypt

**Keywords:** Atrial fibrillation, Cerebrovascular stroke, Multislice computed tomography

## Abstract

**Background:**

Atrial fibrillation (AF) affects millions of people worldwide and can remain undiagnosed for years. It is a major cause of cerebrovascular stroke (CVS); hence, early detection is extremely important in order to decrease the risk of CVS. We conducted a retrospective observational study looking into the prevalence of silent AF in 3299 patients admitted from January 2014 to December 2017 in a tertiary care stroke specialized center. Ischemic CVS was confirmed either by using multislice computed tomography (MSCT) or magnetic resonance imaging (MRI) of the brain. AF was diagnosed by electrocardiography (ECG) at the time of admission or during the hospital stay. Patients with a history of AF were excluded from the study.

**Results:**

Of the 3299 patients admitted by acute ischemic CVS, 707 (21.43%) had a history of AF and thus were excluded from the study. Of the remaining 2592 patients eligible for the study, 1666 (64.27%) were males with a mean age of 56.06 years (± 16.01). A total of 2313 (89.24%) patients remained in sinus rhythm throughout the hospital stay, 211 (8.14%) patients were in AF on admission, and 68 (2.62 %) patients developed AF during their hospital stay. The total number of newly diagnosed patients with AF was 279 (10.76%).

**Conclusion:**

The prevalence of de novo atrial fibrillation in patients presented with acute cerebrovascular stroke is high. The implementation of good screening programs can significantly reduce the risk of disabilities and morbidities.

## Background

Atrial fibrillation (AF) is the most common form of cardiac arrhythmia. In 2010, the estimated global age-adjusted prevalence of AF was 0.5%, which equates to 33 million patients, and it is projected to double by 2050 [1] and in other estimations, it will be doubled by 2030 [2]. However, since most of the studies were conducted in the Western countries and there are very few epidemiological studies of AF in the rest of the world, it is very hard to get accurate global estimates of the disease burden [3]. In the USA, it is estimated that the cost related to AF is 6–26 billion dollars per year [4]. Consequently, AF already has a huge impact on the economy and public health. Moreover, since up to 40% of atrial fibrillation (AF) patients are asymptomatic [5]. Therefore, many patients are undiagnosed. Hence, the prevalence is expected to be much higher than the previous numbers.

AF is associated with a significant burden of morbidity and mortality. The disease can be asymptomatic and remain undiagnosed until patients present with an ischemic stroke [6]. Patients diagnosed with AF have a fivefold increased stroke risk [7]. Nearly 25% of all strokes in the elderly population are related to AF [8]. Early diagnosis of AF is very important as the risk of stroke is significantly decreased by oral anticoagulation treatment [9]. Almost one third of all ischemic strokes are cryptogenic, and recent data of new studies support that silent AF, which is an asymptomatic form of AF incidentally detected during a routine examination or manifesting with a complication, is a major contributor to cryptogenic strokes [10, 11]. Stroke without detectable etiology is frustrating for patients, their families, and the caring physician [12].

The purpose of this study was to estimate the prevalence of patients who present with acute ischemic CVS having no history of AF and are found to be in AF at admission or who developed AF during the hospital stay.

## Methods

This study was a retrospective observational analysis of all patients admitted with an acute CVS to a tertiary care stroke specialized center between 1 January 2014 and 31 December 2017. 3299 patients were diagnosed as CVS by either using multislice computed tomography (MSCT) or magnetic resonance imaging (MRI) of the brain on admission. Patients with a history of previous AF, ischemic or hemorrhagic stroke were excluded from the study. After exclusion, 2592 patients out of 3299 patients were eligible for the study.

Patients were monitored via continuous telemetry throughout the hospital stay; AF was diagnosed by electrocardiography (ECG) that was taken at admission or during hospital stay. Risk factors of stroke including hypertension, diabetes mellitus, smoking, coronary artery disease, chronic kidney disease, peripheral arterial disease, and thrombophilia were gathered. In addition to heart rhythm at admission, complete blood count, creatinine level, thyroid-stimulating hormone (TSH) level, and international normalization ratio (INR) were recorded. Clinical vital signs were recorded at the time of admission: blood pressure and heart rate.

Echocardiographic parameters at admission were recorded. Known heritable thrombophilias by history defined as patients with factor V Leiden mutation, prothrombin G20210A mutation, deficiency of protein C or deficiency of protein S were documented [13]. The widely used CHA_2_DS_2_-VASc score was calculated for all patients (congestive heart failure, hypertension, age ≥ 65 years, diabetes mellitus, vascular disease, female gender [1 point for presence of each], and stroke/TIA and age ≥ 75 years [2 points for presence of each]; scores range from 0 to 9) [14].

The study protocol was approved by the Medical Ethics Committee of the Hospital. There was no need for an informed consent from the patients, as it was a retrospective observational study. The study is compatible with the Declaration of Helsinki.

Statistical analyses were performed using IBM SPSS software version 17 and its related materials. Categorical data is presented as frequency and percentages while continuous data is presented as a mean and standard deviation for normally distributed variables or as a median and interquartile range for non-normally distributed variables.

## Results

Of the 3299 patients admitted with acute ischemic CVS, 707 (21.43%) patients were excluded due to a history of previous AF, ischemic or hemorrhagic stroke.

The remaining 2592 (78.56%) patients were eligible for the study, the median age was 56.06 years (± 16.01) and 1666 (64.27%) were males, 1849 patients (71.33%) were hypertensive and 925 patients (35.69%) were diabetic, and 377 patients (14.54%) had a history of coronary artery disease (CAD), whereas 115 patients (4.44%) had a history of peripheral arterial disease (Table 1). The mean CHADS-VASc score of the studied cases was 2 (± 1.62).

**Table 1:** Baseline demographic, clinical data of patients with de novo AF presented with acute ischemic CVS

		***n*** = 2592
**Age (years)**		**56.06 (± 16.01)**
**Sex (males)**		**1666 (64.27%)**
**Hospital stay (days)**		**4.36 (± 3.47)**
**Risk factors**	**Hypertension**	**1849 (71.33%)**
	**Diabetes mellitus**	**925 (35.69%)**
	**Smoking**	**1445 (55.75%)**
	**Coronary artery disease**	**377 (14.54%)**
	**Chronic kidney disease**	**177 (6.83%)**
	**Peripheral arterial disease**	**115 (4.44%)**
	**Thrombophilia**	**32 (1.23%)**
**CHADS-VASc**		**2 (± 1.62)**

Data is shown in numbers (%) or mean (± standard deviation)

**Fig. 1:**
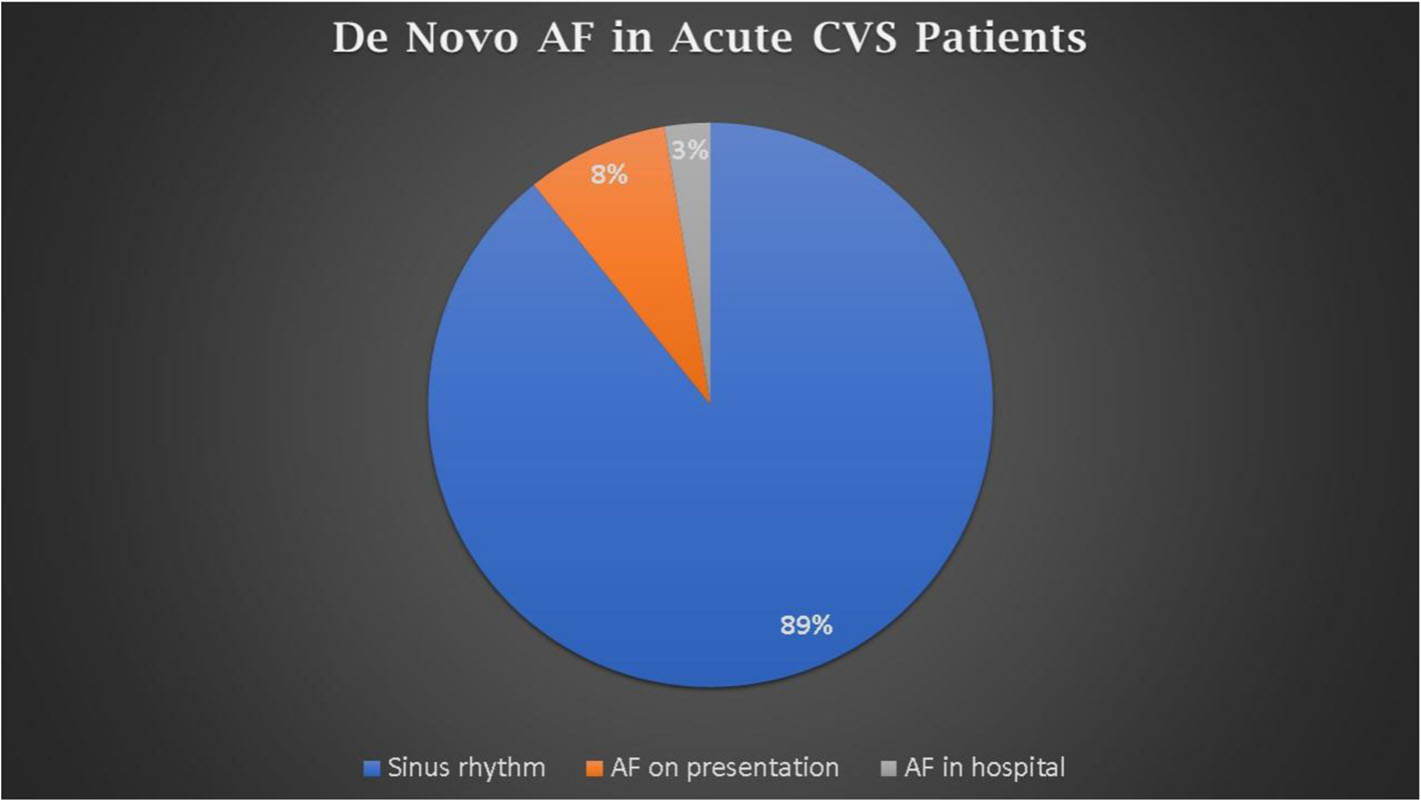
Prevalence of de novo AF in acute ischemic CVS patients

A total of 2313 (89.24%) patients were in sinus rhythm throughout the hospital stay, 211 (8.14%) patients presented with AF rhythm on admission and 68 (2.62 %) patients developed AF during their hospital stay with a cumulative AF prevalence of (10.76%) (Table 2, Fig. 1).

**Table 2:** Baseline laboratory and ECG data of de novo AF patients presented with acute ischemic CVS patients

		***n*** = 2592
**Laboratory data**	**Hemoglobin (g/dl)**	**11.13 (± 1.78)**
	**White cell count (10**^**9**^**/l)**	**8.1 (± 2.13)**
	**Platelet count (10**^**9**^**/l)**	**250 (± 43)**
	**Creatinine (mg/dl)**	**1 (± 0.22)**
	**INR**	**1.03 (± 0.1)**
	**TSH (mIU/L)**	**2.85 (± 1.7)**
**ECG**	**Non-AF**	**2313 (89.24%)**
	**AF on admission**	**211 (8.14%)**
	**AF in hospital**	**68 (2.62 %)**
	**Total AF**	**279 (10.76%)**
**Echocardiography data**	**Ischemic heart disease**	**260 (10.03%)**
	**Valvular heart disease**	**34 (1.31%)**
	**Ejection fraction (%)**	**53.2 (± 19)**
	**LAVI (ml/m2)**	**35 (± 10.3)**
**Clinical examination**	**Mean blood pressure (mmHg)**	**122 ± 15**
	**Heart rate (bpm)**	**82 ± 16**

Data is shown in numbers (%) or mean (± standard deviation)

*AF* atrial fibrillation, *LAVI* left atrial volume index, *INR* international normalization ratio, *TSH* thyroid-stimulating hormone

## Discussion

It has been shown in several studies that silent AF is one of the unrecognized causes of cryptogenic stroke [15]. Diagnosis of silent AF is of great importance in order to decrease ischemic CVS and related morbidity, mortality, disability, and financial loss [16]. Moreover, in patients with a previous cryptogenic stroke, the diagnosis of AF provides the etiology of stroke and generally leads to a change in antithrombotic strategy from antiplatelets to anticoagulation. A recent meta-analysis compared outcomes between patients with asymptomatic and symptomatic AF. The study showed no difference in both general and cardiovascular death risks as well as thromboembolic risk between both groups [5]. Hence, the thromboembolic risk of atrial fibrillation has no relation to symptoms perceived by the patients.

The prevalence of asymptomatic AF depends on the population screened, the device and the duration of monitoring. Consequently, the literature reports a prevalence ranging from 0.5 % in single time point testing, reaching 50% in studies with prolonged continuous ECG monitoring with either surface ECGs or implantable cardiac devices. In a systematic review of AF screening that included 30 studies, single time point pulse palpation or ECG detected 1.4% of undiagnosed AF in adults aged more than 65 years [17]. In the STROKESTOP Study, 7173 Participants without a previous diagnosis of AF underwent intermittent ECG recordings over 2 weeks. 3.0% were found to have AF, and of these, AF was found in 0.5% of the screened population on their first ECG. The use of intermittent ECGs increased new AF detection 4-fold [18]. In the ASSERT study, 2455 participants aged > 65 years with hypertension but no prior history of AF who were receiving a pacemaker. Over a mean 2.5-year follow-up, 34.7% of the patients developed AF. In addition, there was a 2.5-fold increase in the risk of stroke or systemic thromboembolism in patients diagnosed with asymptomatic AF compared to no AF [19]. In a recent study, the estimated prevalence of undiagnosed AF in the USA in 2009 was found to be 700,000 while the total prevalence of AF was 5.3 million, which means 1/8 of all AF patients was undiagnosed. Additional to this data, it was shown that more than half of the population with undiagnosed AF was at moderate to high risk for stroke. The data of this study also showed that the targeted screening strategy may provide benefit especially in the high-risk subgroup of older than 65 years and with multiple CHADS2 risk factors [20]. In a meta-analysis that investigated patients presenting with a stroke or TIA, 7.7% of the patient were diagnosed with atrial fibrillation in the emergency department, with an additional 5.1% during the hospital stay [21]. Our study has supported the previous data showing that the prevalence of undiagnosed AF in patients presented with acute CVS is high given that the monitoring period was just limited to the hospital stay.

Routine pulse self-monitoring in patients over 65 years of age is a class I recommendation in the European Society of Cardiology guidelines for the management of AF to detect silent AF [22]. Despite this high recommendation level, less than 50% of the electrophysiologists who joined the European Heart Rhythm Association survey utilize this method in their daily practice [23].

It is well established that atrial fibrillation increases stroke risk. This risk can be reduced by anticoagulation. Consecutively, several studies investigated the stroke reduction rate after screening high-risk patients and treating them accordingly afterward. In a large United Kingdom (UK) cohort study [15], the included 5555 patients with incidentally detected ambulatory AF were shown to have a high risk of developing CVS; that risk can be significantly reduced by anticoagulation treatment as compared to no therapy.

It is estimated that 1 in every 4 patients presenting with stroke or TIA will be diagnosed with AF if systematic, long-term screening is performed [21]. Therefore, after TIA or stroke, most guidelines recommend screening patients for the presence of AF, with 12-lead ECG, Holter monitoring, telemetry, or monitoring devices, but the exact timing and duration of screening with these techniques are undefined. A meta-analysis showed that 1-week ECG monitoring is cost-effective in the diagnosis of silent AF in order to prevent ischemic events recurrence in patients with ischemic stroke [24]. The difficulty is to extend the monitoring opportunity to other groups of patients with AF risk factors. It is obvious that long-term monitoring and routine control of high-risk patients is helpful for detecting silent AF. But unfortunately, the question about cost-effectiveness has no clear answer. To be able to answer all these questions, more investigation and data are needed.

Interest in the issue of detecting silent AF is increasing day by day. However, technology support continues to be one of the most popular topics of recent times.

In a Canadian study, 184 primary care physicians were provided with a KardiaMobile ECG device (AliveCor) for 3 months. Physicians were asked to obtain a single 30-sec ECG recording of all patients seen in their daily practice who were ≥ 65 years old and not previously diagnosed with AF. A total of 7585 patients were screened. AF was detected in 6.2% of the patients [25]. In the Apple Heart Study, 419,297 participants using smart watches with optical sensors were enrolled. Over a median of 117 days of monitoring, 2161 participants (0.52%) received notifications of an irregular pulse. The probability of receiving an irregular pulse notification was low. Among participants who received notification of an irregular pulse, 34% had atrial fibrillation on subsequent ECG patch readings and 84% of notifications were concordant with atrial fibrillation [26]. The results are very promising as with advances in technology diagnosing silent AF could be accomplished with technology that we use on a daily basis.

A limitation of the study is its retrospective nature. However, we enrolled a high number of patients in 4 years, which is like a reflection of real life. Silent AF is not a rare condition, and implementation of good screening programs and new technology can significantly reduce the risk of CVS with appropriate primary prevention by oral anticoagulation therapy. In addition, early detection can decrease the number of disabilities, morbidity, and mortality. However, cost-effectiveness remains to be an unanswered question. We hope that there will be an improvement in silent AF diagnosis and related problems with the help of increasing studies, data, awareness and progressive technology in the near future.

## Conclusion

Our study showed that the prevalence of de novo atrial fibrillation in patients presented with acute cerebrovascular stroke is high and the implementation of good screening programs can significantly reduce the risk of disabilities and morbidities.

## Data Availability

The datasets used and/or analyzed during the current study are available from the corresponding author on reasonable request
